# Wogonoside induces depalmitoylation and translocation of PLSCR1 and N‐RAS in primary acute myeloid leukaemia cells

**DOI:** 10.1111/jcmm.13481

**Published:** 2018-01-29

**Authors:** Hui Li, Xiaoxuan Yu, Xiao Liu, Po Hu, Le Shen, Yuxin Zhou, Yu Zhu, Zhiyu Li, Hui Hui, Qinglong Guo, Jingyan Xu

**Affiliations:** ^1^ State Key Laboratory of Natural Medicines Jiangsu Key Laboratory of Carcinogenesis and Intervention Key Laboratory of Drug Quality Control and Pharmacovigilance China Pharmaceutical University Nanjing China; ^2^ Department of Hematology The First Affiliated Hospital of Nanjing Medical University Jiangsu Province Hospital Nanjing China; ^3^ Department of Hematology The Affiliated Drum Tower Hospital of Nanjing University Medical School Nanjing China

**Keywords:** wogonoside, depalmitoylation, translocation, PLSCR1, N‐RAS, acute myeloid leukaemia

## Abstract

Acute myeloid leukaemia (AML) comprises a range of disparate genetic subtypes, involving complex gene mutations and specific molecular alterations. Post‐translational modifications of specific proteins influence their translocation, stability, aggregation and even leading disease progression. Therapies that target to post‐translational modification of specific proteins in cancer cells represent a novel treatment strategy. Non‐homogenous subcellular distribution of PLSCR1 is involved in the primary AML cell differentiation. However, the nuclear translocation mechanism of PLSCR1 remains poorly understood. Here, we leveraged the observation that nuclear translocation of PLSCR1 could be induced during wogonoside treatment in some primary AML cells, despite their genetic heterogeneity that contributed to the depalmitoylation of PLSCR1 via acyl protein thioesterase 1 (APT‐1), an enzyme catalysing protein depalmitoylation. Besides, we found a similar phenomenon on another AML‐related protein, N‐RAS. Wogonoside inhibited the palmitoylation of small GTPase N‐RAS and enhanced its trafficking into Golgi complex, leading to the inactivation of N‐RAS/RAF1 pathway in some primary AML cells. Taken together, our findings provide new insight into the mechanism of wogonoside‐induced nuclear translocation of PLSCR1 and illuminate the influence of N‐RAS depalmitoylation on its Golgi trafficking and RAF1 signalling inactivation in AML.

## Introduction

Acute myeloid leukaemia (AML) is a clinically devastating disease characterized by the arrest of leukaemic blast at the early stages of differentiation and the retention of self‐renewal stage during leukaemic myeloblast development 1. With the development of karyotyping and genetic analysis, the genetic heterogeneity of AML was observed, comprising a range of genetic subtypes 2. Even with progress in diagnosis and supportive care, the survival rate and prognosis are dismal 1. AML cases involve complex gene mutations and heterogeneous combinations of chromosomal alterations 3, such as mutations or abnormal expressions of N‐RAS, FLT3, CEBPA, PLSCR1 and so on 3, 4, 5, 6, 7, highlighting the difficulty in illuminating the pathogenesis of AML and developing specific therapy targets and drugs. Molecular analysis of common genetic alterations in leukaemic cells has contributed greatly to our understanding of the pathogenesis of AML 8. Drugs targeting the key molecules of AML cells are likely to provide a new therapeutic strategy.

Reagents targeting the post‐translational modification of critical pathogenic proteins in AML malignancy, including the farnesyltransferase inhibitor (FTI), ubiquitin–proteasome inhibitor and histone deacetylase inhibitor (HDACI), have been the focus of intense interest for drug development. Thereinto, palmitoylation is a post‐translational lipid modification involving the attachment of a 16‐carbon saturated fatty acid, palmitate, to cysteine residues of substrate proteins *via* a labile thioester bond 9. Reversible attachment and removal of palmitate from proteins have been suggested to control diverse biological processes 10, 11, 12. The palmitoylation status of protein allows it to be attached to the specific plasma membrane compartments or transported into the nucleus, controlling the activation and functions of proteins 13, 14. Moreover, dynamic cycles of palmitoylation/depalmitoylation of proteins influence their trafficking, stability and aggregation 9. Increasing evidence suggested that the intervention of palmitoylation modification of specific proteins is a feasible and effective strategy for the treatment of AML 15, 16. Therefore, efforts are underway to find new drugs that control the post‐translational modification of critical pathogenic proteins in AML malignancy.

Previous investigation demonstrated that phospholipid scramblase 1 (PLSCR1) expression was up‐regulated by wogonoside in 14 of 23 (~61%) of patients with AML [PBMC derived from four relapsed (RR) and 19 newly diagnosed (ND) patients were analysed for PLSCR1 expression using Western blots], suggesting that PLSCR1 acts as an important effector in wogonoside‐treated primary AML cells 17. Multiple lines of investigation have revealed that AML‐M1, ‐M5a and ‐M5b possess lower PLSCR1 expression compared to normal bone marrow (BM) cells 7. Besides, higher PLSCR1 mRNA levels showed better outlook for overall survival in patients with AML 7, reflecting the critical function of PLSCR1 in the development of disparate AML subtypes. Putting aside the development of genetic subsets, PLSCR1 expression of AML cases can be used to assess a patient's disease progression and outcome. Further evidences uncovered that the nuclear translocation of PLSCR1 is essential to the differentiation of AML cells 17, 18. Despite this, there is a limited understanding of the mechanism for nuclear translocation of PLSCR1 in primary AML cells. PLSCR1 itself is a multipalmitoylated plasma membrane protein, whose palmitoylation/depalmitoylation status acts as a switch to control its own destination to either the plasma membrane or the nucleus 13. Previous research has revealed that PLSCR1 transported to the nucleus after cytokine stimulation 13, 19. Inspired by these findings, we reasoned that the palmitoylation/depalmitoylation status of PLSCR1 might contribute to its subcellular localization and function in wogonoside‐treated AML cells. Moreover, wogonoside exerts potent palmitoylation modification activity in primary AML cells.

According to the hypothesis that wogonoside may control the palmitoylation status of PLSCR1, thus facilitating its attachment to the specific plasma membrane compartments or nuclear transportation, we speculated that the effect of wogonoside on palmitoylation modification might be non‐specific. The small GTPase RAS is usually used as a paradigm to show how reversible acylation allows proteins to be trapped and released from specific membrane compartments. The mutational activation of *Ras* gene and high expression of oncogenic RAS proteins commonly exist in approximately 30% of human cancers, including myeloid malignancies 20, 21. Recent analyses of cancer genomes have confirmed the central role of *Ras* as a driver of pathogenesis in several human tumours 22. There are three major RAS isoforms, H/N/K‐RAS, of which K‐RAS and N‐RAS mutation activation are prevalent in AML patient samples 3, 21, 23. Especially, the N‐RAS is the most commonly affected in leukaemia, with activating mutations occurring in around 20% of AML specimens 23. It is well known that RAS proteins undergo a complex series of post‐translational processing steps, including farnesylation and palmitoylation 9, 24. Of which, palmitate turnover controls the localization of RAS and regulates the RAS signalling 25, 26. Frequently, RAS was served as the paradigm of proteins' palmitoylation/depalmitoylation modifications 9. The function of RAS for signalling is inextricably linked to its enrichment at plasma membrane where the RAS is highly palmitoylated 27. Activated and guanine triphosphate (GTP)‐loaded RAS recruits effector proteins such as RAF kinase to the plasma membrane, thereby initiating signalling cascades that result in cell proliferation and survival 28, 29, 30, 31. Therefore, the amount of RAS that resides on the plasma membrane is crucial for signal output. The N‐RAS isoform is reversibly palmitoylated on one or two cysteines in the hypervariable region (HVR). Post‐translational modifications make N‐RAS lipophilic and therefore enable its association with membranes 32. Moreover, N‐RAS palmitoylation/depalmitoylation cycles could lead to N‐RAS/RAF1 pathway inactivation and is associated with the distribution from the plasma membrane towards the Golgi 33, 34. Beyond these observations, an unmet challenge is to develop novel therapeutic strategies for AML treatment, such as the specific N‐RAS post‐translational modification‐targeting drugs.

Our previous studies have demonstrated the antileukaemic properties of wogonoside, a flavonoid extracted from *Scutellaria baicalensis Georgi* (huang qin), both *in vivo* and *in vitro*, and highlighted the nuclear translocation of PLSCR1 in wogonoside‐induced primary AML cell differentiation 17, 18. However, the mechanism underlying wogonoside‐induced nuclear translocation of PLSCR1 remains elusive to date. Here, our findings further clarify the key regulation effect of wogonoside on protein palmitoylation modification in disparate primary AML cells, raising a potential therapeutic approach for treating a range of genetic AML subtypes.

## Materials and methods

### Reagents and antibodies

Wogonoside (≥98% purity; Langze Pharmaceutical Co, Ltd, Nanjing, China) was dissolved in dimethylsulphoxide (DMSO) as a stock solution at 0.5 M and stored at −20°C. Wogonoside stock solution was freshly diluted with medium to the final concentration (150 μM) before each experiment. The final DMSO concentration did not exceed 0.1%. Cells treated with the highest concentration of DMSO were used as control in the corresponding experiments.

All‐trans retinoic acid (ATRA) was dissolved in DMSO as a stock solution at 0.01 M and used as an inducer of PLSCR1. A nuclear/cytosol fractionation kit (KeyGEN Biotech, Nanjing, China) was used according to the manufacturer's directions. *N*‐Ethylmaleimide (NEM), hydroxylamine (HAM) solution, 4, 6‐diamidino‐2‐phenylindole dihydrochloride (DAPI), MTT [3‐(4,5‐dimethylthiazol‐2‐yl)‐2,5‐diphenyl tetrazolium bromide] and cycloheximide (CHX) were purchased from Sigma‐Aldrich (St. Louis, MO, USA). Biotin‐BMCC, Streptavidin‐HRP and Western blot Stripping Buffer were purchased from Thermo Scientific (Waltham, MA, USA).

PLSCR1 (Human) IP‐WB antibody pair and APT1 antibody were purchased from Abnova (Taiwan, China). Primary antibody against β‐actin, laminA, N‐RAS and APT1 siRNA were obtained from Santa Cruz Biotechnology (Santa Cruz, CA). Fluorescein isothiocyanate (FITC) anti‐human CD14 was obtained from Miltenyi Biotec Inc (Auburn, CA, USA). Phycoerythrin (PE) anti‐human CD11b antibodies were obtained from eBioscience (San Diego, CA, USA). Antibody against RAF1 and p‐RAF1 were products from Abclonal Biotechnology (Nanjing, China). Anti‐GM130 antibody was obtained from BD Biosciences (Cambridge, UK). IRDyeTM 800‐conjugated secondary antibodies were purchased from Rockland (Philadelphia, PA, USA). Alexa Fluor^®^ 488 goat anti‐rabbit IgG (H+L) antibody, Alexa Fluor^®^ 488 donkey anti‐goat IgG (H+L) antibody and Alexa Fluor^®^ 555 donkey antimouse IgG (H+L) antibody were purchased from Life Technologies (Carlsbad, CA, USA).

### Cell culture

Human AML cell line U937 and HL‐60 were from Cell Bank of Shanghai Institute of Biochemistry & Cell Biology. Primary leukaemic cells from newly diagnosed AML patients without prior therapy (The First Affiliated Hospital of Nanjing Medical University, Nanjing, China) were collected using lymphocyte–monocyte separation medium (Jingmei, Nanjing, China). The protocol of collection of cells from patients complied with guidelines in the Declaration of Helsinki and was approved by The First Affiliated Hospital of Nanjing Medical University's institutional review board and the appropriate ethics committees. A signed informed consent was obtained from each patient. Primary leukaemia cell isolation was performed as described previously in Hussong *et al*. 35. U937, HL‐60 and primary leukaemic cells were cultured in RPMI‐1640 medium, supplemented with 10% FBS, 100 U/ml of benzylpenicillin and 100 μg/ml of streptomycin in a humidified environment with 5% CO_2_ at 37°C.

### Immunoprecipitation and acyl‐biotin exchange (IP‐ABE) assay

ABE assay is the most ideal method for measuring the dynamic turnover of palmitate on proteins 36. Cells were collected, and unmodified cysteine thiol groups of lysates were blocked irreversibly using N‐ethylmaleimide (NEM). Protein was purified *via* IP 37. Specific cleavage and unmasking of the palmitoylated cysteine thiol group was performed with hydroxylamine (HAM). Meanwhile, groups with omission of HAM cleavage were prepared as a negative control. Subsequently, we used biotin‐BMCC to selectively label palmitoylated cysteine. Palmitoylation of PLSCR1 was detected using a streptavidin–horseradish peroxidase (streptavidin–HRP) antibody, and HRP luminescence exposed using a chemiluminescent substrate kit. These results were photographed using a Bio‐Rad phosphorimager and analysed with Image Lab™ Software, Version 3.0 (Bio‐Rad, Hercules, CA, USA). Finally, protein expression was measured using Western blot. Detection was performed with the Odyssey Infrared Imaging System (LI‐COR Inc, Lincoln, NE, USA). Palmitoylation of PLSCR1 was quantified using image analysis software, and relative palmitoylation levels normalized to those of immunoprecipitated protein.

### Transient transfection with small interfering RNA (siRNA)

Cells were plated in six‐well plates with fresh RPMI 1640 medium. siRNA transfection was performed with Lipofectamine 2000 reagent according to the manufacturer's instructions 38.

### Differentiation assay

Fluorescence intensity of CD11b and CD14 was analysed with a FACSCalibur flow cytometer (Becton‐Dickinson, San Jose, CA, USA) 39. Data were based on the examination of 10,000 cells per sample selected randomly from 5 × 10^5^ cells.

### Preparation of cytosolic and nuclear extracts

Primary AML cells were collected at various time‐points after wogonoside (150 μM) treatment. A nuclear–cytosol fractionation kit used the nuclear and cytosolic protein extraction preparations according to the modified method as described 40. The cytosolic and nuclear fractions were subjected to Western Blot analysis.

### Western blot analysis

Preparation of whole‐cell lysates was performed as described previously 37. Then equal amounts of extracts (50 μg) were separated by 8–12% sodium dodecyl sulphate–polyacrylamide gel electrophoresis (SDS‐PAGE) and transferred onto the PVDF membranes (Millipore, Boston, MA, USA). The blots were incubated with specific antibodies overnight at 4°C followed by IRDyeTM800‐conjugated secondary antibody for 1 hr at 37°C. Detection was performed with the Odyssey Infrared Imaging System.

### Immunofluorescence

Cells were collected onto the coverslips and fixed in ice‐cold methanol for 10 min. Then coverslips were permeabilized in 0.2% (v/v) Triton X‐100 for 20 min. and blocked with BSA buffer (PBS containing 3% BSA) for 1 hr at room temperature. Next, cells were co‐incubated with GM130 (1:100) and primary anti‐N‐RAS antibody (1:50) and then 4°C overnight. The coverslips were washed three times and followed by co‐incubation with Alexa Fluor^®^ 555 Donkey antimouse IgG (H+L) antibody (1:500) and Alexa Fluor^®^ 488 Goat anti‐Rabbit IgG (H+L) antibody (1:500) for 1 hr at 37°C. Then, the coverslips were washed and counterstained with DAPI working solution (100 μg/ml) for 20 min. at room temperature. The coverslips were inverted onto slides and immersed in a mounting medium. The images were captured with a confocal microscope at 1000× magnification (FV1000; Olympus, Tokyo, Japan).

### Statistical analysis

All data were expressed as mean ± SD from at least three independent experiments performed in a parallel manner. Statistical analysis of multiple‐group comparisons was performed by one‐way analysis of variance (anova) followed by the Bonferroni *post hoc* test. Comparisons between two groups were analysed using two‐tailed Student's *t*‐tests. A *P* value < 0.05 was considered statistically significant.

## Results

### Wogonoside facilitates the nuclear distribution of PLSCR1 in primary AML cells

**Figure 1:**
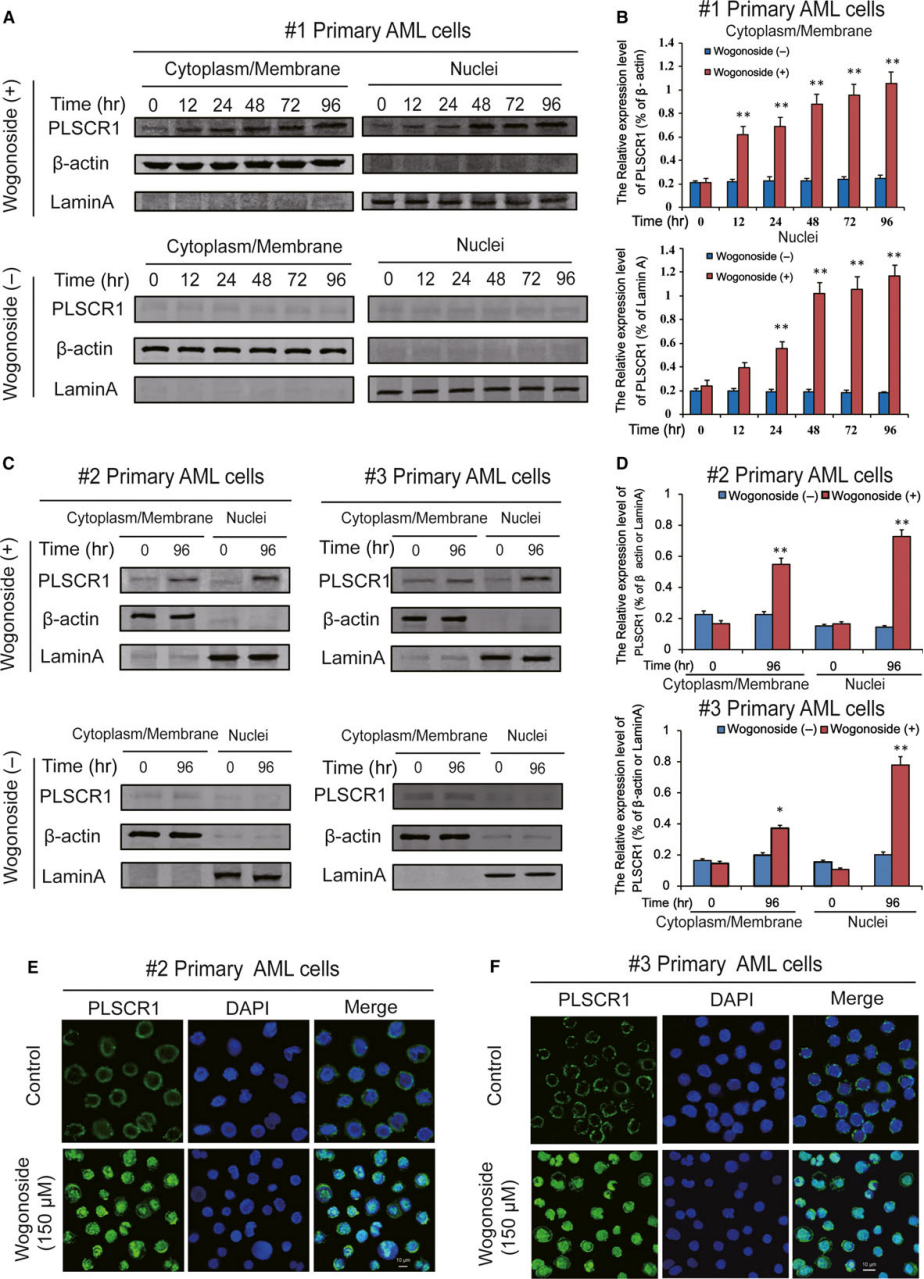
Nuclear distribution of PLSCR1 in different wogonoside responder's primary AML patient samples. **(B)** Cytoplasmic/membrane and nuclear fractions of the wogonoside responder′s cells (#1) were analysed by Western blot for the PLSCR1 protein, with β‐actin and laminA as cytoplasmic and nuclear loading controls, respectively. Each protein band was derived from different gels. (**B**) Data represent the mean ± SEM from three independent experiments. Asterisks denote statistically significant (*P <* *0.05 and **P <* *0.01) differences compared with controls by one‐way anova. (**C**) The other two wogonoside responder′s samples (#2 and #3) were analysed by Western blot for the nuclear translocation of PLSCR1, with β‐actin and laminA as cytoplasmic and nuclear loading controls, respectively. (**D**) Data represent the mean ± SEM from three independent experiments. Asterisks denote statistically significant (*P <* *0.05 and **P <* *0.01) differences compared with controls by one‐way anova. (**E**,** F**) Immunofluorescence of 150 μM wogonoside‐treated primary AML cells (#2 and #3) for 96 hrs costained with anti‐PLSCR1 (primary)/Alexa Fluor^®^ 488‐labelled donkey anti‐goat (secondary) antibody combinations (green fluorescence), as well as DAPI (blue fluorescence), to visualize the nuclei. They were detected by confocal microscopy (FV1000; Olympus, Tokyo, Japan) with FV10‐ASW2.1 acquisition software (Olympus) at room temperature (Original magnification ×1000; immersion objective ×100 with immersion oil type F). Images are representative of 3 independent experiments.

**Figure 2:**
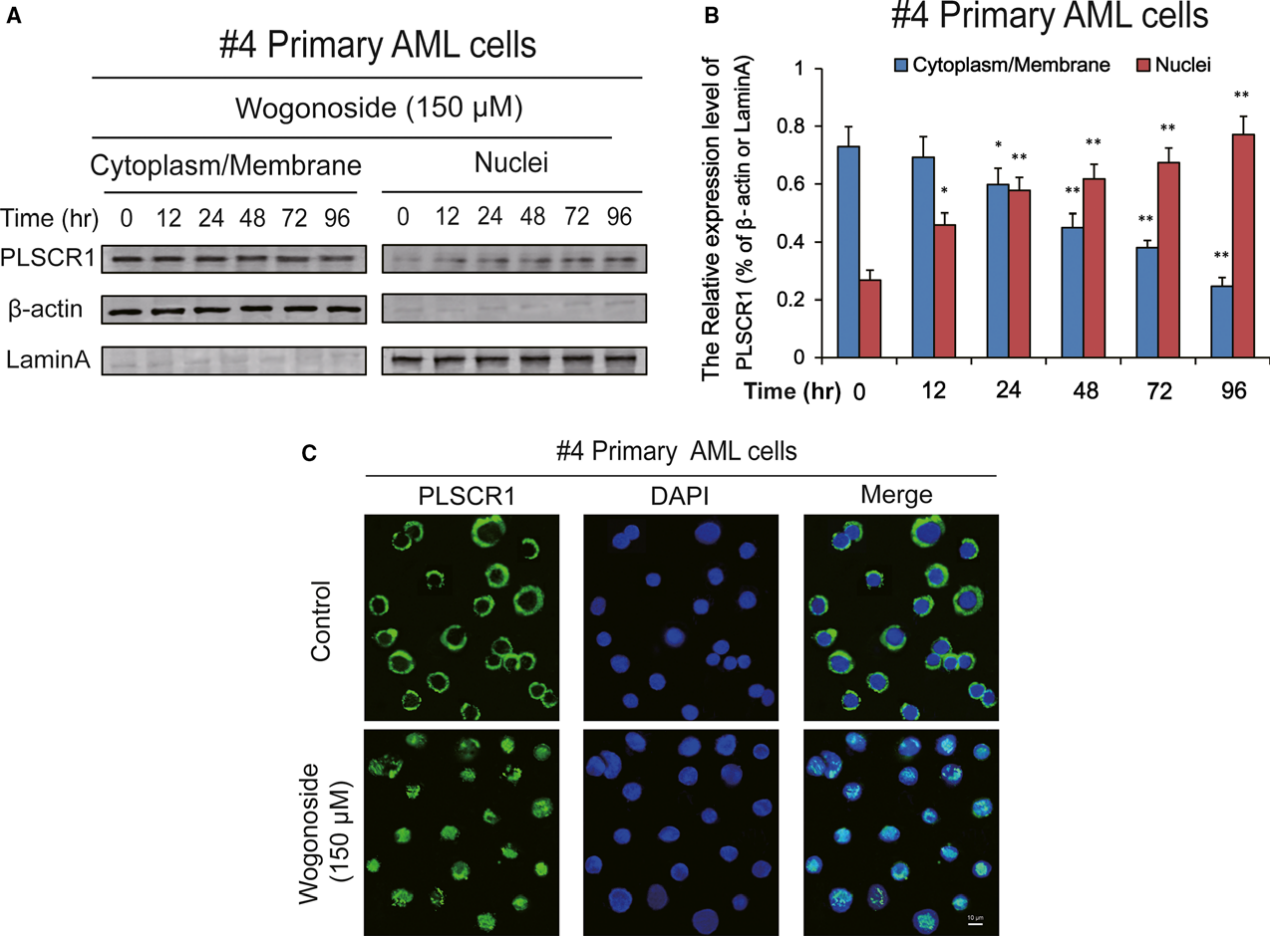
Nuclear distribution of PLSCR1 in wogonoside non‐responder′s primary AML patient sample. (**A**) The cytoplasmic/membrane and nuclear fractions of the PLSCR1^high^ but wogonoside non‐responder′s cells (#4) were analysed by Western blot for the PLSCR1 nuclear translocation, with β‐actin and laminA as cytoplasmic and nuclear loading controls, respectively. (**B**) Data represent the mean ± SEM from three independent experiments. Asterisks denote statistically significant (*P <* *0.05 and **P <* *0.01) differences compared with controls by one‐way ANOVA. (**C**) Immunofluorescence of 150 μM wogonoside‐treated #4 primary AML cells for 96 hrs costained with anti‐PLSCR1 (primary)/Alexa Fluor^®^ 488‐labelled donkey anti‐goat (secondary) antibody combinations (green fluorescence), as well as DAPI (blue fluorescence), to visualize the nuclei. They were detected by confocal microscopy (FV1000; Olympus, Tokyo, Japan) with FV10‐ASW2.1 acquisition software (Olympus) at room temperature (Original magnification ×1000; immersion objective ×100 with immersion oil type F). Images are representative of three independent experiments.

Measuring of wogonoside‐induced myeloid differentiation is typically performed in AML cell lines, and the mechanism of differentiation is not clearly defined. We have used a range of AML samples to confirm that PLSCR1 expression and its nuclear function are critical to the differentiation of primary AML cells 17. To further verify the effects of wogonoside on PLSCR1 nuclear translocation in primary AML cells, we firstly evaluated the subcellular distribution of PLSCR1 in primary cells from four clinical AML samples (Table 1). PLSCR1 expression in primary AML samples (#1, #2, and #3) was low but can be significantly increased by wogonoside. Next, nuclear and cytoplasm/membrane proteins of #1 primary AML cells were fractionated in the presence of wogonoside for 0, 12, 24, 48, 72, and 96 hrs. Wogonoside promoted the nuclear translocation of PLSCR1 starting at 24 hrs and reached a plateau at 48 hrs in primary AML cells, while PLSCR1 was scarcely detected in the nuclei of control cells (Fig. 1A,B). Meanwhile, we observed that wogonoside treatment increased the expression of PLSCR1 in cytoplasm/membrane, demonstrating the expected effects of wogonoside on PLSCR1 expression (Fig. 1A,B). Similar to the results of samples #1, the nuclear translocation of PLSCR1 was confirmed by nuclear/plasma protein separation assay in #2 and #3 primary AML cells after treated with wogonoside for 96 hrs (Fig. 1C,D). Furthermore, as shown in Figure 1E,F, we observed that PLSCR1 was mostly distributed in the membrane before wogonoside treatment using immunofluorescence confocal microscopy, and wogonoside (150 μM) could significantly induce nuclear translocation of PLSCR1 at 96 hrs. In addition, we further found that the background expression of PLSCR1 of sample #4 was high but unaffected by wogonoside. Notable nuclear translocation of PLSCR1 was also observed in sample #4 (Fig. 2A,B). The nuclear expression of PLSCR1 was increased in #4 primary AML cell treated with wogonoside for 0, 12, 24, 48, 72 and 96 hrs, resembling #1 primary AML cells. Immunofluorescence confocal microscopy assay also proved the phenomenon of wogonoside‐induced nuclear translocation of PLSCR1 in #4 primary AML cell (Fig. 2C). However, unlike #1, #2 and #3 samples, the cytoplasmic/membrane expression of PLSCR1 was continually decreased in sample of #4, suggesting that wogonoside may only affect nuclear translocation, but not the total expression of PLSCR1 in #4 primary AML cells (Fig. 2).

**Table 1:** Clinical data for patient samples with AML

Patient no.	Diagnosis	Source	PB blast %	BM blast %	WBC	FAB	Cytogenetics	Status
#1	AML	PB			331.08	M1	CD34‐ANLL	New
#2	AML	PB	88		29.5	M1	CEBPA mutation; BCR‐ABL(9;22)(+)	New
#3	AML	PB		87.5	43.36	M5a	CBFβ/MYH11(+); BCR/ABL(−)	New
#4	AML	PB			31.0	M1	OD	New
#5	AML	PB	92			M1	OD	New
#6	AML	PB	82	4.8	81.5	M2b	FLT‐ITD	New

PB, peripheral blood; BM, bone marrow; WBC, white blood cells count; FAB, French‐American‐British; OD, outside diagnosis.

### Wogonoside‐induced nuclear distribution of PLSCR1 is independent of the increasing expression of PLSCR1 in primary AML cells

**Figure 3:**
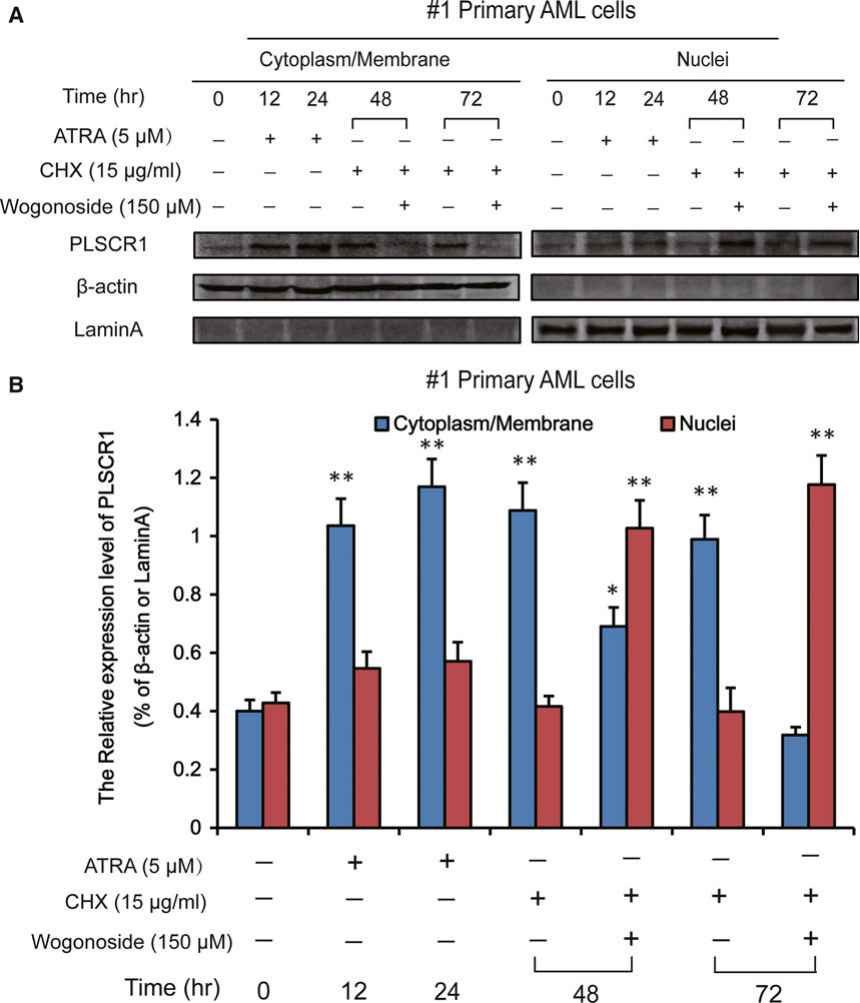
Wogonoside‐induced nuclear distribution of PLSCR1 is independent of PLSCR1 expression up‐regulation in #1 primary AML cell. (**A**) Effects of wogonoside on nuclear translocation of PLSCR1 in #1 primary AML cells in the presence of ATRA and CHX. #1 Primary AML cells were incubated with ATRA (5 μM) for indicated time. ATRA was removed at 48 hrs, and then cells were pre‐treated with CHX (15 μg/ml) for 1 hrs, followed by 150 μM wogonoside for 24 hrs and 48 hrs, respectively. The cytoplasmic/membrane and nuclear fractions of the cells were analysed by Western blot for the subcellular distribution of PLSCR1 in the absence or presence of 150 μM wogonoside, with β‐actin and laminA as cytoplasmic and nuclear loading controls, respectively. Each protein band was derived from different gels. (**B**) Data represent the mean ± SEM from three independent experiments. Asterisks denote statistically significant (*P <* *0.05 and **P <* *0.01) differences compared with controls by one‐way anova.

Given that wogonoside (150 μM) treatment not only increased the expression of PLSCR1 but also promoted its translocation to nucleus in primary AML cells (#1, #2 and #3), we were intrigued whether the nuclear translocation of PLSCR1 was due to the up‐regulation of PLSCR1 expression. To further ascertain this reasoning, #1 primary AML cells were pre‐treated with ATRA for 48 hrs, and then, the cells were pre‐treated with 15 μg/mL cycloheximide (CHX), the protein synthesis inhibitor, followed by 150 μM wogonoside for 24 hrs and 48 hrs. ATRA induced the up‐regulation of PLSCR expression in a time‐dependent manner without distribution priority, suggesting that ATRA can elevate PLSCR1 expression but cannot promote its nuclear translocation in primary AML cells (Fig. 3A). Moreover, wogonoside‐induced PLSCR1 nuclear translocation keeps increasing after incubation with CHX, indicating that the nuclear import of PLSCR1 in primary AML cells exposed to wogonoside was not due to PLSCR1 synthesis enhancement (Fig. 3B).

### Effects of wogonoside on depalmitoylation of PLSCR1 in AML cell lines and primary AML cells

**Figure 4:**
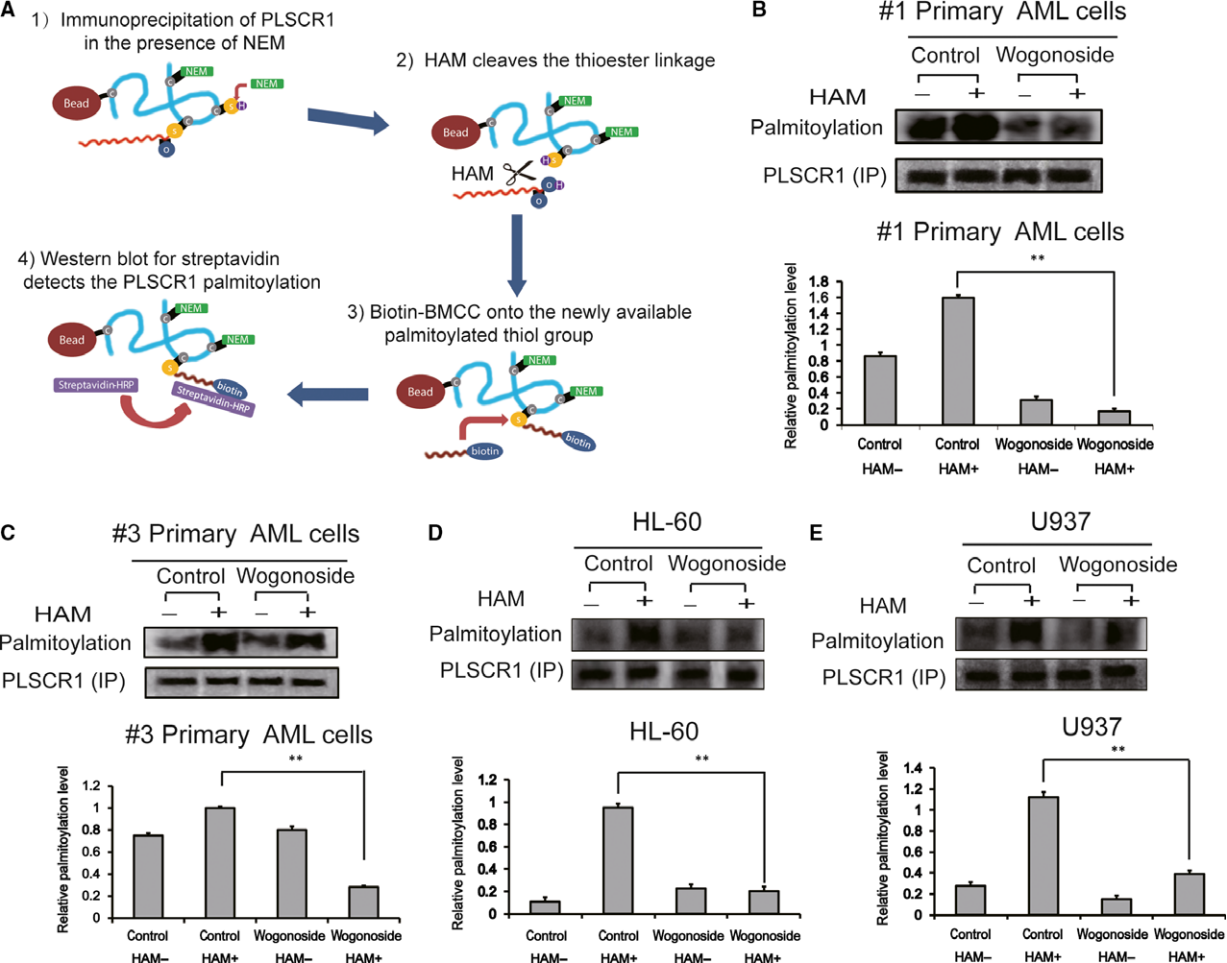
Wogonoside induces depalmitoylation of PLSCR1 in AML cell lines and primary AML cells. (**A**) Schematic of the Immunoprecipitation and acyl‐biotin exchange (IP‐ABE) assays that purify and detect palmitoylation of PLSCR1. (**B**,** C**) Primary AML cells (#1 and #3) were incubated with or without wogonoside (150 μM) for 48 hrs, and PLSCR1 expression (IP) and depalmitoylation effect were determined using IP‐ABE assays. The relative palmitoylation level of PLSCR1 in primary AML cells is shown. Data represent mean ± SEM from three independent experiments. Asterisks denote statistically significant (**P <* *0.01) differences compared with controls by one‐way anova. (**D**,** E**) The palmitoylation and expression of PLSCR1 in HL‐60 and U937 cells were analysed by IP‐ABE assays after wogonoside treatment for 48 hrs. The relative palmitoylation level of PLSCR1 in U937 and HL‐60 cells is shown. Data represent mean ± SEM from three independent experiments. Asterisks denote statistically significant (**P <* *0.01) differences compared with controls by one‐way anova.

PLSCR1 is a multipalmitoylated protein, and previous research has revealed that a significant fraction of PLSCR1 is depalmitoylated 13. Palmitoylation of PLSCR1 acts as a switch, controlling destination of PLSCR1 to either the plasma membrane or the nucleus 13. Therefore, we reasoned that the nuclear import of PLSCR1 in primary AML cells exposed to wogonoside was more likely to be attributed to PLSCR1 depalmitoylation. The immunoprecipitation–acyl‐biotin exchange (IP‐ABE) assay facilitated specific detection of thiol‐palmitoylated cysteine residues along the immunoprecipitated PLSCR1 protein (Fig. 4A). To determine the mechanism underlying wogonoside's activity on the localization and nuclear translocation of PLSCR1, we indirectly detected the relative palmitoylation levels of PLSCR1 in primary AML cells (samples #1 and #3) (Fig. 4B,C) and AML cell lines including U937 and HL‐60 (Fig. 4D,E) treated with wogonoside (150 μM) for 48 hrs using IP‐ABE assay. We purified PLSCR1 in primary AML cells by immunoprecipitation assay using an antibody directed against PLSCR1 and measured equal PLSCR1 level in each group. Western blotting results demonstrated that the purified PLSCR1 protein is comparable in each group of AML cells (Fig. 4B–E). According to the ABE assay to directly measure palmitoylation level of the PLSCR1, it is evident that wogonoside markedly decreased the palmitoylation of the purified PLSCR1 upon exposure to HAM, resulting in specific cleavage of thioester bonds at palmitoylated cysteines and the unmasking of a free palmitoylated thiol group (‐SH) (Fig. 4B–E). Meanwhile, we found that the palmitoylation was detectable by Western blotting with streptavidin–HRP (palmitoylation) in the minus‐hydroxylamine (HAM) groups of #1 and #2 primary AML cells (Fig. 4B,C). These results might be due to the competing attachment between biotin–BMCC and a 16‐carbon saturated fatty acid, palmitate, to cysteine residues of substrate proteins. So far, we have confirmed that wogonoside decreased the palmitoylation levels of PLSCR1 in U937, HL‐60 and primary AML cells, suggesting the nuclear trafficking of PLSCR1 was more likely to result from its depalmitoylation.

### APT1 knock‐down suppresses the effects of wogonoside on the depalmitoylation and nuclear translocation of PLSCR1 in primary AML cells

**Figure 5:**
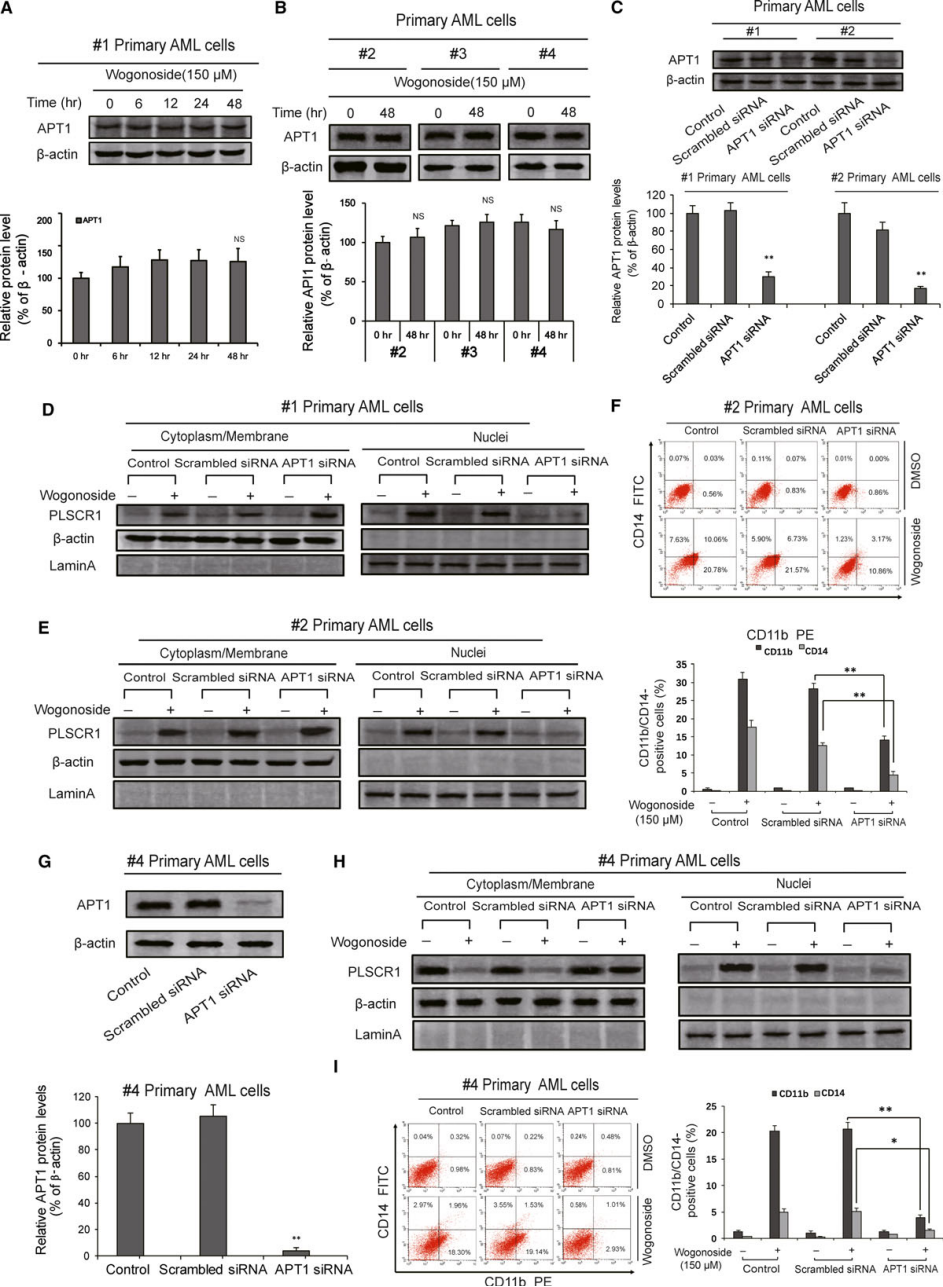
APT1 is involved in wogonoside‐induced nuclear trafficking of PLSCR1 and cell differentiation in different primary AML cells. (**A**,** B**) APT1 expression levels of primary AML cells (#1, #2, #3 and #4) were analysed by Western blotting after treatment with wogonoside (150 μM) for indicated time. β‐actin was used as a loading control. Data represent mean ± SEM from three independent experiments. Asterisks denote statistically significant (**P <* *0.01) differences compared with controls by one‐way ANOVA. (**C**,** G**) #1, #2 and #4 primary AML cells were transfected with non‐specific siRNA and APT1 siRNA for 48 hrs. Confirmation of the knock‐down of APT1 expression was detected by Western blotting with β‐actin as a loading control. Results are representative of three independent experiments. (**D, E, H**) Shown are the effects of the knock‐down APT1 on the nuclear translocation of PLSCR1, which could be influenced by 150 μM wogonoside, with β‐actin and laminA as cytoplasmic and nuclear loading controls, respectively. Results are representative of three independent experiments. (**F, I**) Shown are the effects of knock‐down APT1 on wogonoside‐induced differentiation in primary AML cells (#2 and #4). CD11b and CD14 expression of primary AML cells were detected by flow cytometry analyses. CD11b‐ and CD14‐positive ratio of primary AML cells is shown; columns represent means of 3 different experiments; bars represent standard errors.

Acylprotein thioesterase‐1 (APT1) is a 29‐kDa cytosolic protein that exhibits lysophospholipase activity towards palmitoylated glycerol‐3‐phosphocholine and can remove palmitate from proteins on the cytosolic surface of membranes 9. As APT1 might be a key contributor to wogonoside‐induced depalmitoylation of PLSCR1, we analysed the expression level of APT1 in wogonoside‐treated AML primary cells from #1, #2, #3 and #4 samples. Our results showed no significant alteration in APT1 expression after 150 μM wogonoside treatment for 0, 6, 12, 24 and 48 hrs in #1 primary AML cells (Fig. 5A), or for 48 hrs in #2, #3 and #4 primary AML cells (Fig. 5B), suggesting that APT1 expression could be irrelevant to wogonoside‐induced nuclear translocation of PLSCR1. We therefore knocked down APT1 by siRNA in #1 and #2 primary AML cells and used Western blotting to monitor the efficiency of transfection (Fig. 5C). As the results show, 69.77% and 83.05% of APT1 expression in #1 and #2 primary AML cells were knocked down, respectively (Fig. 5C). Knock‐down of APT1 reversed the nuclear translocation of PLSCR1 in these two cell types, indicating that nuclear trafficking of PLSCR1 induced by wogonoside required APT1 activity (Fig. 5D,E). In #4 sample, the nuclear translocation of PLSCR1 was also dramatically inhibited (Fig. 5H) upon 96.35% of APT1 expression were knocked down (Fig. 5G). To further investigate whether the differentiation‐promoting effect of wogonoside on primary AML cells is dependent on APT1 expression, cells (sample #2 and #4) were transfected with APT1 siRNA. Cell differentiation analyses were subsequently performed using FACS assays. Notably, upon knock‐down of APT1, wogonoside‐induced differentiation effects on primary AML cells were significantly reduced. In primary cells from samples #2, wogonoside‐induced up‐regulation of CD11b and CD14 was decreased to a certain extent (Fig. 5F). Moreover, we obtained that the expression of CD11b and CD14 was essentially unchanged in #4 primary AML cells (Fig. 5I). Hence, our data supported the key role of APT1 in wogonoside‐induced nuclear translocation of PLSCR1 in primary AML cells.

### Wogonoside‐induced depalmitoylation and Golgi translocation of N‐RAS are dependent on APT1 in primary AML cells

**Figure 6:**
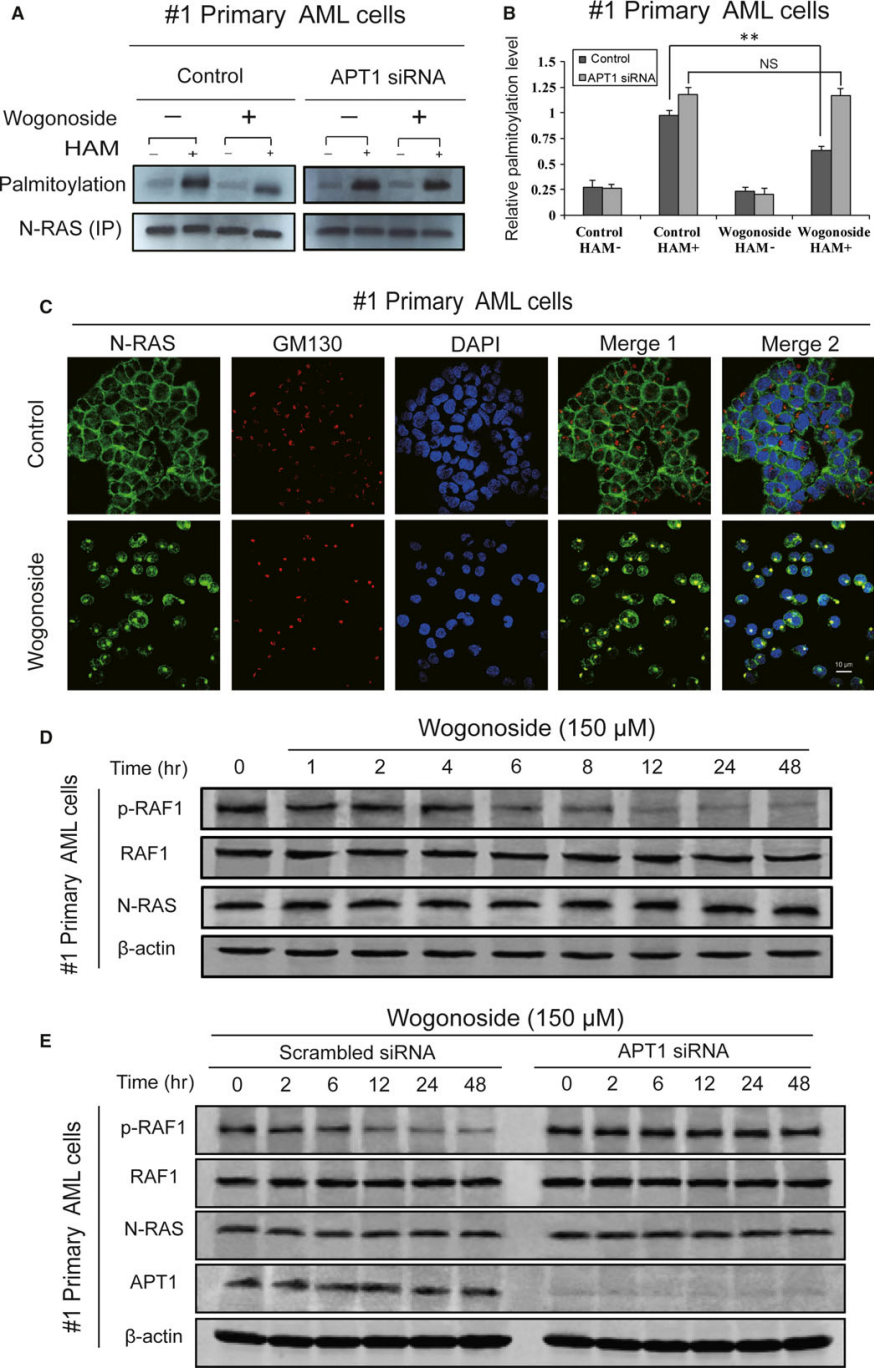
Effects of wogonoside on N‐RAS in primary AML cells. (**A**) #1 primary AML cell was transfected with APT1 siRNA treated with or without 150 μM wogonoside for 48 hrs, and the palmitoylation level of N‐RAS was detected by IP‐ABE assay. (**B**) The data represent the mean ± SEM of 3 different experiments. Asterisks denote statistically significant (*P <* *0.05 and **P <* *0.01) differences compared with controls by one‐way ANOVA. (**C**) Immunofluorescence of 150 μM wogonoside‐treated #1 primary AML cells for 48 hrs was performed. Cells were collected and were costained with anti‐GM130 (primary)/Alexa Fluor^®^ 555 Donkey antimouse (secondary) antibody (red fluorescence) combinations anti‐N‐RAS (primary)/Alexa Fluor^®^ 488 Goat anti‐Rabbit (secondary) antibody (green fluorescence), as well as DAPI (blue fluorescence). They were detected by confocal microscopy (FV1000; Olympus, Tokyo, Japan) with FV10‐ASW2.1 acquisition software (Olympus) at room temperature (Original magnification ×1000; immersion objective ×100 with immersion oil type F). Images are representative of 3 independent experiments. (**D**) #1 primary AML cells were cultured for 0, 1, 2, 4, 6, 8, 12, 24 and 48 hrs with 150 μM wogonoside. Whole‐cell extracts at different time‐points were analysed by Western blotting for expression levels of N‐RAS, RAF1 and p‐RAF1, using β‐actin as a loading control. The data represent the mean ± SEM of 3 different experiments. (**E**) #1 primary AML cells were transfected with non‐specific siRNA and APT1 siRNA treated with or without 150 μM wogonoside for 0, 2, 6, 12, 24 and 48 hrs. Whole‐cell extracts at different time‐points were analysed by Western blotting for expression levels of APT1, N‐RAS, RAF1 and p‐RAF1, using β‐actin as a loading control. The data represent the mean ± SEM of three different experiments.

From the results above, we found that wogonoside promoted the depalmitoylation of PLSCR1 *via* APT1 in AML cell lines and primary AML cells. We speculated that the effect of wogonoside on post‐translational modification might be non‐specific. It is well known that N‐RAS is a palmitoylated and hyperactivated kinase in AML 23, 24. Next, we detected the effect of wogonoside on palmitoylation of N‐RAS. #1 primary AML cell was treated with or without 150 μM wogonoside for 48 hrs. Results demonstrated that wogonoside markedly down‐regulated the palmitoylation level of N‐RAS (Fig. 6A,B). Furthermore, with APT1 knock‐down, wogonoside‐induced depalmitoylation of N‐RAS was impaired in #1 primary AML cell (Fig. 6A,B). In addition, membrane localization of N‐RAS is believed to directly determine the activation of RAS signalling 27. Activated N‐RAS recruits effector proteins such as RAF kinase to the plasma membrane, thereby initiating signalling cascades and controlling the fate of the AML cells. Moreover, a literature search revealed that the presence of reversibility of palmitoylation and depalmitoylation on N‐RAS affects either the attachments of N‐RAS to membrane compartment or its trafficking to Golgi. To further confirm wogonoside‐induced N‐RAS trafficking, we next investigated the localization of N‐RAS in primary AML cells (#1, #5 and #6) after treatment of 150 μM wogonoside for 48 hrs. Results showed that wogonoside significantly facilitated the Golgi translocation of N‐RAS (Fig. 6C, Figure S1, Table 1).

The activation of small GTPase RAS and its kinase cascade RAF1 contributes to a number of cellular processes including cell proliferation, survival, differentiation and apoptosis 29, 41. In particular, the N‐RAS/RAF1 pathway plays a key role in haematopoietic malignancies 21. To certify whether the phosphorylation activation of RAF1 in primary AML cells could be inhibited after depalmitoylation of N‐RAS induced by wogonoside, we next detected the expression of p‐RAF1 during the palmitoylation decrease in N‐RAS. #1 primary AML cells were treated with wogonoside for 0, 1, 2, 4, 6, 8, 12, 24 and 48 hrs. Phosphorylation of RAF1 was analysed by Western blotting. Results demonstrated that N‐RAS and RAF1 expression were not affected by wogonoside. However, the phosphorylation of RAF1 was gradually decreased in a time‐dependent manner (Fig. 6D). In addition, the activation ability of N‐RAS to downstream RAF1 was reduced upon APT1 knock‐down in samples #1, and RAF1 continued to be activated after the translocation of N‐RAS was blocked (Fig. 6E). Taken together, the results suggested that wogonoside facilitated the depalmitoylation of N‐RAS and promoted its Golgi trafficking, which in turn inhibited RAF1 phosphorylation and eventually caused the inactivation of N‐RAS/RAF1 pathway.

## Discussion

Although the contribution of PLSCR1 nuclear translocation to wogonoside‐induced differentiation of AML cells has been reported 17, 18, the trafficking mechanism and molecular bases remained unclear. Moreover, previous studies highlighted the role of palmitoylation modification and membrane localization of RAS in the activation of RAS/RAF1 pathway 27, 28, 29, 30. However, no drug has been developed so far by targeting the palmitoylation status of N‐RAS in AML cells. Here, we demonstrated that wogonoside‐induced translocation of critical proteins by depalmitoylation modification.

The palmitoylation state has been known to determine the subcellular localization of PLSCR1, and depalmitoylated PLSCR1 can be imported into the nucleus for binding genomic DNA 13, 19, 42. Indeed, depalmitoylation of PLSCR1 was observed after treatment with wogonoside for 48 hrs in U937, HL‐60 and primary AML cells (Fig. 4) when expression of PLSCR1 was significantly up‐regulated, indicating that depalmitoylation might be the actual cause of nuclear translocation of PLSCR1. Most importantly, wogonoside induced the nuclear trafficking of ATRA‐induced PLSCR1 when protein biosynthesis was inhibited by CHX, suggesting the ability of wogonoside to initiate nuclear localization of PLSCR1 in a way independent of its transcriptional up‐regulation effect. Apart from samples (#1, #2 and #3) with low background PLSCR1 expression, we further observed increased PLSCR1 nuclear translocation induced by wogonoside in sample (#4) with high background PLSCR1 expression, although its expression level was barely affected by wogonoside. These data further confirmed that wogonoside could initiate nuclear translocation of PLSCR1 independently of its up‐regulation.

Protein palmitoylation and depalmitoylation have been proven to be a reversible process regulated by two types of enzymes, acyltransferases (PATs) and protein thioesterases (APT1 and PPT1) 43. APT1 can remove palmitate from proteins on the cytosolic surface of membranes, leading to the dynamic subcellular trafficking of proteins such as H‐Ras 44, whereas PPT1 is a soluble lipase that is localized in lysosomes and responsible for the depalmitoylation of proteins during protein degradation 9. Wogonoside showed no effect on expression of APT1, but APT1 knock‐down reversed wogonoside‐induced nuclear translocation of PLSCR1, proving the essentiality of APT1 in wogonoside‐induce depalmitoylation and nuclear translocation of PLSCR1. We conjecture that wogonoside may affect the enzyme activity of APT1, but the selectivity of proteins in wogonoside‐induced depalmitoylation effects remains to be further elucidated.

Multiple lines of research on the regulation of RAS focused on the post‐translational modifications, including the constitutive and irreversible remodelling of its carboxy‐terminal CAAX motif by farnesylation, proteolysis and methylation, reversible palmitoylation and conditional modifications 24. Palmitoylation of N‐RAS protein increased its affinity for phospholipid bilayers required for its binding to the plasma membrane and entry to specific membrane trafficking pathways. More recently, the palmitoylation/depalmitoylation cycle of N‐RAS was shown to be causally linked to bidirectional trafficking between the Golgi and the plasma membrane 33, 44. Studies on RAS trafficking in mammalian cells have confirmed a model in which an acylation cycle regulates the localization of RAS and its access to regulators and effectors 9. Our data showed that wogonoside promoted the depalmitoylation of N‐RAS and accelerated its Golgi translocation in primary AML cells. Moreover, previous studies demonstrated that the downstream activation effect of N‐RAS depends on its traffic to plasma membrane compartments 29. Therefore, we detected the subcellular localization of N‐RAS after the treatment of 150 μM wogonoside for 48 hrs. Results showed that wogonoside could effectively stimulate the Golgi translocation of N‐RAS. In addition, wogonoside significantly inhibited the activation of N‐RAS/RAF1 pathway. The dwell time of palmitoylated RAS on the inner leaflet of plasma membrane is regulated by depalmitoylation 26. We thus conclude that wogonoside triggers depalmitoylation and thereby limits the residence of N‐RAS at the plasma membrane. The current strategy of developing new drugs that limit N‐RAS activity mostly focuses on the inhibition of farnesylation. However, the clinical efficacy of FTIs is disappointing 45, 46. In the light of these results, it was expected that new drug development focusing on the inhibition of the palmitoylation modification would prevent membrane localization of oncogenic RAS and thereby inhibit its activity. Therefore, wogonoside‐induced depalmitoylation of N‐RAS represents a promising approach in AML treatment. Moreover, data acquisition on the basis of patient‐derived samples ensures that our findings comply with many clinical observations in individuals with AML. Besides, MTT test of primary AML cells and human normal peripheral blood mononuclear cells (PBMC) showed that wogonoside had no obvious effect on the cell viability of PBMC, but could significantly inhibit the growth of AML cells at the same concentration (Figure S2). Our data implied that wogonoside might have a certain selectivity when it exerts antileukaemic effect.

PLSCR1 and N‐RAS signalling play important roles in the progression of AML malignancy. Our previous investigation focused on the effect of wogonoside on the PLSCR1 in 23 AML clinical samples. Samples were divided into three types according to their different responses to wogonoside in PLSCR1 expression 17. In view of the above results, we found that in a type of these primary cells, the background expression of PLSCR1 was low and did not respond to wogonoside. Similarly, wogonoside showed little differentiation induction effect on this type of cells. To sum up, we found that PLSCR1 is not suitable for all types of samples as a target of the wogonoside, but PLSCR1 plays an important role in the process of differentiation induced by wogonoside. N‐RAS activation is ubiquitous in AML cells, so the dual effects of wogonoside on PLSCR1 and N‐RAS have extend the scope of effect of wogonoside on various types of AML to a certain extent. The effect of wogonoside on N‐RAS mainly affects the growth and proliferation of AML cells and the subsequent apoptotic pathway. The effect of wogonoside on the palmitoylation of these two proteins induced different subsequent effects, which is beneficial to exert the anti‐AML effect of wogonoside.

Targeting the post‐translational modifications of these proteins is a new strategy for the design and development of anti‐AML drugs. This study demonstrated that wogonoside promotes both PLSCR1 nuclear translocation and Golgi trafficking, which are responsible for its antileukaemia activity, by inducing PLSCR1 depalmitoylation and N‐RAS depalmitoylation, respectively. These findings suggest the potential role of wogonoside as a novel agent in AML treatment.

## Author contributions

H.L. designed and performed research, analysed data and wrote the manuscript; X.Y. and X.L. performed research. P.H. and L.S. analysed data; Y.Z. performed the animal experiments; J.X. and H.H. collected data and performed statistical analysis; Y.Z. provided the blood samples; Z.L. analysed the compound; Q.G. and J.X. conceptualized the project and directed experiment design and data analysis.

## Conflict of interest

The authors declare no conflict of interest.

## Supporting information


**Figure S1** (A, B) Immunofluorescence of 150 μM wogonoside‐treated primary AML cells (#5 and #6) for 48 hrs was performed. Cells were collected and were co‐stained with anti‐GM130 (primary)/Alexa Fluor® 555 Donkey anti‐Mouse (secondary) antibody (red fluorescence) combinations anti‐N‐RAS (primary)/Alexa Fluor® 488 Goat anti‐Rabbit (secondary) antibody (green fluorescence), as well as DAPI (blue fluorescence). They were detected by confocal microscopy (FV1000; Olympus, Tokyo, Japan) with FV10‐ASW2.1 acquisition software (Olympus) at room temperature (Original magnification ×1000; immersion objective ×100/×60 with immersion oil type F). Images are representative of three independent experiments.

**Figure S2** Growth inhibition effects of wogonoside on AML cells and human normal peripheral blood mononuclear cells (PBMC). Primary AML cells (#1, #2, and #3) and PBMC were incubated in 96‐well plates with 5 × 10^4^/well in 100 μL culture medium, then were treated with 100 μL various concentration of wogonoside for 96 hrs, respectively. Cell viability was determined using MTT assay. Data were shown as means ± SD (*n* = 3).
