# Supplementary material for: Wogonoside induces depalmitoylation and translocation of PLSCR1 and N‐RAS in primary acute myeloid leukaemia cells
Source: J Cell Mol Med. 2018 Jan 29;22(4):2117–30. doi: 10.1111/jcmm.13481 (PMC5867108; doi:10.1111/jcmm.13481)
Supplement: Supplementary file 3 [file JCMM-22-2117-s003.docx]

**Supplementary Figure 1:** (A, B) Immunofluorescence of 150 μM wogonoside-treated primary AML cells (#5 and #6) for 48 h was performed. Cells were collected and were co-stained with anti-GM130 (primary)/ Alexa Fluor® 555 Donkey anti-Mouse (secondary) antibody (red fluorescence) combinations anti-N-RAS (primary)/ Alexa Fluor® 488 Goat anti-Rabbit (secondary) antibody (green fluorescence), as well as DAPI (blue fluorescence). They were detected by confocal microscopy (FV1000; Olympus, Tokyo, Japan) with FV10-ASW2.1 acquisition software (Olympus) at room temperature (Original magnification ×1000; immersion objective ×100/×60 with immersion oil type F). Images are representative of 3 independent experiments.

**Supplementary Figure 2: Growth inhibition effects of wogonoside on AML cells and human normal peripheral blood mononuclear cells (PBMC).** Primary AML cells (#1, #2, and #3) and PBMC were incubated in 96-well plates with 5×10^4^/well in 100 μL culture medium, then were treated with 100 μL various concentration of wogonoside for 96 h, respectively. Cell viability was determined using MTT assay. Data were shown as means ± SD (n = 3).
